# Effect of nitrogen supply on nitrogen metabolism in the citrus cultivar ‘Huangguogan’

**DOI:** 10.1371/journal.pone.0213874

**Published:** 2019-03-21

**Authors:** Ling Liao, Tiantian Dong, Xinya Liu, Zhixiang Dong, Xia Qiu, Yi Rong, Guochao Sun, Zhihui Wang

**Affiliations:** 1 College of Horticulture, Sichuan Agricultural University, Chengdu, China; 2 Institute of Pomology and Olericulture, Sichuan Agricultural University, Chengdu, China; Hainan University, CHINA

## Abstract

Nitrogen metabolism in citrus has received increased attention due to its effects on plant growth and productivity. However, little is known about the effects of nitrogen fertilization on nitrogen metabolism in young trees of citrus cultivar ‘Huangguogan’ (*Citrus reticulata × Citrus sinensis*). Here, genes encoding nitrate reductase (NR), nitrite reductase (NiR), glutamine synthetase (GS), glutamate dehydrogenase (GDH), and asparagine synthetase (AS), represented as *HgNR*, *HgNiR*, *HgGS*, *HgGDH*, and *HgAS*, respectively, were cloned from Huangguogan. Deduced protein sequences were analyzed and proteins were confirmed to be localized in their respective cellular organelles. Moreover, pot-cultured ‘Huangguogan’ seedlings were fertilized with 0 (N_1_), 1.36 (N_2_), 1.81 (N_3_), 2.26 (N_4_), or 2.72 (N_5_) kg N/year, for 12 months. Enzyme activity and enzyme-gene expression were studied in roots, leaves, and fruits at different stages. Finally, the effects of N application rate on root activity, leaf N, soluble protein, yield, and fruit quality at the ripening stage were measured. The results showed that: 1) *HgNR*, *HgNiR*, *HgGDH*, and *HgAS* gene products were found mainly in the cytoplasm and plasma membrane, while *HgGS* gene product was found mainly in cytoplasm and mitochondria. 2) Gene expression and enzyme activity differed among plant organs. As the root is in permanent direct contact with the soil we suggest that root gene expression and enzyme activity can be used as reference to determine N application rate. 3) Yield, fruit quality, enzyme activity, and enzyme-related gene expression were considerably lower at low than at high-N supply. However, they were all inhibited by excess nitrogen (i.e., 2.72 kg/year). Therefore, we recommend 1.81 kg N/year as the optimal N application rate for young ‘Huangguogan’ trees.

## Introduction

Citrus are important fruits worldwide. China has the largest area of cultivated citrus and the total national production ranks third in the world. The vegetative development of citrus trees is dependent on nutrient availability [1]. Among all the nutrients involved in plant metabolism, N is a major limiting factor for plant production [2] and an essential structural constituent of proteins, RubisCO, nucleic acids, and chlorophyll, in addition to some hormones [3]. N fertilizer plays a vital role in citrus tree growth, fruit yield and quality, as shown by several reports on the effects of N application rate on citrus growth [4,5]. Sufficient levels of N support regular plant growth and help plants to defend against stress [6–8]. However, N deficiency leads to poor plant growth [9], small fruit size [10], reduced photosynthetic capacity [11], and production [12]. It has been proposed that under drought or water stress, crops show better growth performance at relatively higher N [6,8,13] rates, while excessive use of N leads to nutritional imbalance, cell membrane destruction, and oxidative stress; further, excess N causes severe environmental damage, N loss, and costs increase to farmers [14–17]. Annual application rates of N fertilizers have recently increased dramatically in intensive agricultural systems in China, frequently resulting in excess application [18]. Therefore, a reduction in the use of nitrogen fertilizers is required that does not impact crop productivity [19].

Nitrogen metabolism is one of the basic processes of plant physiology that controls many cellular activities in plants [20] and is crucial for stress tolerance [21]. Plants absorb N either as nitrate or ammonium, and then convert these to various amino acids [22]. Therefore, the activities of N assimilating enzymes play a significant role in maintaining growth and development [23]. Nitrate reductase (NR), nitrite reductase (NiR), glutamine synthetase (GS), glutamate dehydrogenase (GDH), and asparagine synthetase (AS) are all key enzymes involved in N metabolism, whose activities have been used as representative biochemical markers to evaluate plant N status [24]. Nitrate (NO_3_^−^) reduction to NH_4_^+^ is mediated by nitrate NR and NiR; NH_4_^+^ is then converted by GS to glutamine and in turn levels of α-ketoglutarate are equilibrated by GDH activity. Finally, glutamate together with oxaloacetate can be used to generate aspartate and asparagine by the sequential action of aspartate aminotransferase and AS [5].

NR is the most important rate-limiting enzyme for N assimilation, and NR activity is used as an indicator of plant N status and requirements by citrus trees under orchard cultivation conditions [25,26]. It has been shown that an appropriate amount of N increased NR activity in different vegetative organs [27]. NiR is regulated transcriptionally, usually in coordination with NR, to avoid nitrite toxicity. For this reason, cells must contain enough NiR to reduce all the nitrite produced by NR [5]. A previous report showed that NiR was predominant and at similar levels in leaf and fruit tissues in citrus [28]. NiR and NR are regulated by gene expression in a similar manner, and their overexpression resulted in a reduction in nitrate levels in plant tissues [29]. GS is a multifunctional enzyme at the center of N metabolism, which participates in the regulation of various N metabolism-related reactions and is induced by increased NH_4_^+^ supply [30]; GS activity has been shown to be significantly and positively related to the ability to adapt to abiotic stress [31]. Indeed, GS activity reportedly declined, leading to a significant decrease in N metabolism-related enzyme activity, thereby affecting the synthesis and transformation of amino acids [32,33]. It has been also reported that the expression of GS increased in roots following treatment with nitrate or ammonium ions, and that overexpression of GS significantly improved in terms of biomass or seed yield [34]. GDH is the main enzyme for primary N assimilation, as its main role is in the deamination of glutamate to provide energy and to return carbon skeletons from amino acids to the reactions of carbon metabolism [35]. For its part, GDH plays an initial role in the synthesis of NH_4_^+^ and a complementary role in the synthesis route of glutamic acid, which is abundant in several plant organs[35]. The level of AS activity is reportedly affected by urea concentration [36] and plays an important role in N recycling via NH_4_^+^ under environmental stress [37]. Many studies have shown that the activity of N metabolism enzymes were significantly reduced under drought, salt, and heavy metal stress [38,39], Liu et al. [40] suggested that GS and GDH activities increased in response to N addition under low-temperature stress; Zhang et al. [41] suggested that NR, GS, and GDH activities were gradually reduced by nitrate stress, thus inhibiting N metabolism in cucumbers. However, little attention has been paid to the effects of N supply on N metabolism in citrus trees. To better understand the underlying physiological mechanisms of N metabolism in response to N levels and how these can be manipulated is essential to improve citrus N metabolism capacity.

‘Huangguogan’ (*Citrus reticulata* × *C*. *sinensis*) is a new, late maturing, high-yielding, seedless, hybrid citrus cultivar in China [42]. Previous studies on ‘Huangguogan’ are scarce, and there has been no research on the effects of N fertilizer on this cultivar. Rather, previous research on N metabolism has mainly focused on barley [43], wheat [44], rice [45], and persimmon [46]. However, there is a dearth of reports pertaining to the role of N in N metabolism of citrus plants. Therefore, we analyzed whether N supplementation modulates N metabolism. Among the various essential enzymes involved in the N metabolism process, we monitored NR, NiR, GS, GDH, and AS by comparing the expression patterns of the corresponding genes, namely *HgNR*, *HgNiR*, *HgGS*, *HgGDH*, and *HgAS*, respectively. We confirmed the subcellular localization of the five enzymes and measured fruit quality. Our results showed that the expression of key genes and enzymes involved in N metabolism were significantly responsive to the amount of N applied, and ultimately, we propose an optimal N application rate for best seedling growth of the citrus cultivar ‘Huangguogan’.

## Materials and methods

### Ethics statement

The study was approved by the peoples’s Government of Shimian, Sichuan,China.

### Plant materials

The field study was conducted on a sandy loam at the Standard Cultivation Demonstration Garden of ‘Huangguogan’ in Shimian County, Sichuan Province, China (29.23°N, 102.36°E; 780 m a.s.l.). Average annual precipitation at this location is 780 mm, and the average annual temperature from March 2017 to April 2018 was 17.1°C (http://www.shimian.gov.cn/htm/about.htm?id=79E11D75-75AD-41E8-A672-C14B6F115B18). Ten-year-old, healthy ‘Huangguogan’ trees grafted onto Trifoliate Orange (*Poncirus trifoliata* L. Raf) were selected. The experiment was laid in a randomized complete block design with five N treatments, each with five replicates. All plants were fertilized using 1.45 kg phosphorus (CaP_2_H_4_O_8_, P_2_O_5_ ≥ 12%) and 2.12 kg potassium fertilizer (K_2_SO_4_, K_2_O ≥ 50.0%) per year. Nitrogen fertilizer in the form of urea [CO(NH_2_)_2_, N ≥ 46.67%] was applied at 0 (N_1_), 1.36 (N_2_), 1.81 (N_3_), 2.26 (N_4_), or 2.72 (N_5_) kg/year at germination (G), physiological fruit dropping (P), young fruit expansion (Y), and in the color-change period (C). In accordance with the sugar-increasing and acid-decreasing methods for citrus cultivar ‘Shimian Huangguogan’ [47], the G:P:Y:C ratios were: 40:10:40:10 for CO(NH_2_)_2_ application, 30:10:40:20 for CaP_2_H_4_O_8_ application, and 20:30:40:10 for K_2_SO_4_ application (Table 1). All trees received normal horticultural care for pest and disease control during the experiment. For each N treatment, the roots, leaves, and fruits were sampled six times, at 60-day intervals, from 60 days to 360 days after blossom. Roots less than 2 mm in diameter were collected from a depth of 0–40 cm in a 40- to 60-cm radius around the trunk of each tree. A total of 40 leaf and 40 fruit samples were collected in four directions from each replicate. All trees sampled had completely developed the third or fourth leaf from the top of the canopy. Samples were immediately frozen in liquid N and stored at −80°C until use for analysis of key enzyme activities, gene expression, and subcellular localization of N metabolism-related gene products.

**Table 1:** Amount of fertilizer (kg/plant) in each growing period.

Treatment	Period	CO(NH_2_)_2_ (N ≥ 46.67%)	CaP_2_H_4_O_8_(P_2_O_5_ ≥ 12%)	K_2_SO_4_(K_2_O ≥50.0%)
N_1_	G	0	0.435	0.424
	P	0	0.145	0.636
	Y	0	0.58	0.848
	C	0	0.29	0.212
N_2_	G	0.544	0.435	0.424
	P	0.136	0.145	0.636
	Y	0.544	0.58	0.848
	C	0.136	0.29	0.212
N_3_	G	0.724	0.435	0.424
	P	0.181	0.145	0.636
	Y	0.724	0.58	0.848
	C	0.181	0.29	0.212
N_4_	G	0.904	0.435	0.424
	P	0.226	0.145	0.636
	Y	0.904	0.58	0.848
	C	0.226	0.29	0.212
N_5_	G	1.088	0.435	0.424
	P	0.272	0.145	0.636
	Y	1.088	0.58	0.848
	C	0.272	0.29	0.212

Fertilization was applied at four stages of development: germination (G); physiological fruit-dropping (P); young fruit expansion (Y) and fruit color-change (C); G:P:Y:C ratios were: 40:10:40:10 for CO(NH_2_)_2_ application, 30:10:40:20 for CaP_2_H_4_O_8_ application, and 20:30:40:10 for K_2_SO_4_ application.

*Nicotiana benthamiana* seeds were germinated in small pots filled with peat moss and grown under controlled conditions at 25°C, 60–70% RH, 4000 lux, and a 14/10 h light/dark regime for 6 weeks.

### Bacterial strains

*Escherichia coli* DH5α and *Agrobacterium tumefaciens* strain GV3101 were used. *Escherichia coli* DH5α and Agrobacterium tumefaciens GV3101 were routinely grown in Luria-Bertani (LB) media containing the appropriate antibiotics at 37 and 30°C, respectively.

### Cloning of *HgNR*, *HgNiR*, *HgGS*, *HgGDH*, and *HgAS* genes

To verify and clone the cDNA sequences of *HgNR*, *HgNiR*, *HgGS*, *HgGDH*, and *HgAS*, total RNA was extracted from ‘Huangguogan’ leaves using RNAiso Plus (TaKaRa, Dalian, China). First strand cDNA was synthesized from total RNA using the PrimeScript^TM^ RT reagent kit (TaKaRa) according to the instructions by the manufacturer. Primers (Table 2) for *HgNR*, *HgNiR*, *HgGS*, *HgGDH*, and *HgAS* genes from the cultivar ‘Huangguogan’ were designed from sequences of *Citrus sinensis NR*, *NiR*, *GS*, *GDH*, and *AS* genes (*Citrus sinensis*: XM_006472645.2, *Citrus sinensis*: XM_006487044.2, *Citrus sinensis*: XM_006489176.2, *Citrus sinensis*: XM_015530679.1, and *Citrus sinensis*: XM_006488487.2). PCRs were set up using 10-μL volumes containing 5 μL Taq RCR Mix (TaKaRa), 1 μL cDNA from ‘Huangguogan’ leaves, 0.5 μL PCR forward primer, 0.5 μL PCR reverse primer and 3 μL RNase-free H_2_O (Tiangen, Beijing, China). PCRs were run under the following cycling conditions: 95°C for 5 min, followed by 36 cycles of 95°C for 30 s, Tm (52.4°C, 53.3°C, 52.1°C, 51.6°C, and 53.8°C, respectively) for 30 s, 72°C for 1 min, and a final extension for 10 min at 72°C. PCR products were detected by 1.5% agarose gel electrophoresis and recovered using an E.Z.N.A Gel Extraction Kit (Omega Bio-Tek, Winooski, VT, America). The products of gel extraction purification were cloned into pMD19-T vector (TaKaRa) according to standard protocols and transformed into *E*. *coli* DH5α. Positive amplicons were confirmed by colony PCR. Six independent positive colonies carrying an insert of the expected size were selected for plasmid purification (Omega) and sequenced by LiuHe HuaDa Biotechnology (Beijing) Co., Ltd. (Beijing, China).

**Table 2:** Primers used for analysis of the expression of *HgNR*, *HgNiR*, *HgGS*, *HgGDH*, and *HgAS*.

Primer	Sequence Fragment	Length(bp)	Melting temperature(°C)
*HgAS*-F	CACTTTTGCCGTCAAACTGACC	1952	53.8
*HgAS*-R	TGTCTTAAGTTCACCCACTCTTGG		
*HgGDH*-F	CGAAGCTGTAATCAGGTTAAG	1403	51.6
*HgGDH*-R	CGGAAGTGAATAAGGGCTCT		
*HgGS*-F	TTTCAGGGAGGAGTAGGTG	1485	52.1
*HgGS*-R	GCTCTCAAGTTCTTCAGTTG		
*HgNiR*-F	CTTAGCCCCATCCAAGAGTC	1869	53.3
*HgNiR-*R	CTCCCTCAGTACAGCTCCAA		
HgNR-F	CCCTTTTCTTTTGAATCTACA	4135	52.4
*HgNR*-R	ATATGTACAACCTCGGCTGG		

### Analysis of *HgNR*, *HgNiR*, *HgGS*, *HgGDH*, and *HgAS* gene sequences

ORF Finder (http://www.ncbi.nlm.nih.gov/gorf/orfig.cgi) was used to identify open reading frames (ORFs); the nucleotide sequences were translated using NCBI (http://www.ncbi.nlm.nih.gov) and ExPASy (http://www.expasy.org), and the SignaIP 4.1 server (http://www.cbs.dtu.dk/services/SignalP/) was used to determine the location of signal peptide cleavage sites in amino acid sequences. The molecular weight and isoelectric point of predicted proteins were obtained using Compute PI/MW tool of ExPaSy. Transmembrane helices were predicted using TMHMM 2.0 (http://www.cbs.dtu.dk/services/TMHMM-2.0/). The sub-cellular localization of translated proteins was predicted using WoLF-PSORT program and SoftBerry (http://linux1.softberry.com/). Amino acid sequences were aligned using ClustalW and phylogenetic trees were constructed by the neighbor joining method with 1000 bootstrap replicates using MEGA X software.

### Analysis of *HgNR*, *HgNiR*, *HgGS*, *HgGDH*, and *HgAS* gene expression

Transcriptional levels of *HgNR*, *HgNiR*, *HgGS*, *HgGDH*, and *HgAS* were analyzed by RT-qPCR using the 18s gene as an internal control with the primers shown in Table 3. Total RNA was extracted from roots, leaves, and fruits using RNAiso Plus (TaKaRa), and treated with RNase-free H_2_O (Tiangen) according to the instructions by the manufacturer. Data were analyzed using Opticon Monitor software (Bio-Rad). Three technical replicates for one of the three biological replicates were performed for each gene. The 2^–ΔΔCt^ method was used to analyze mRNA expression levels [48].

**Table 3:** Primers used for analysis of the expression of *HgNR*, *HgNiR*, *HgGS*, *HgGDH*, and *HgAS*.

Primer	GenBank accession	Sequence Fragment
HgAS-F	MK073922	CATACTTGCTGTTCTCGGTTGC
*HgAS*-R		CCGTTGATGAGCCAAATAAAAG
*HgGDH*-F	MK073921	GGGAAGATTGTTGCTGTAAGTG
*HgGDH*-R		CGAATCTCCACCACTGAATCCT
*HgGS*-F	MK226159	TTGGATTGGAGGTACTGGGATT
*HgGS*-R		TATGGCGTTTGTTTGTAGGGAT
*HgNiR*-F	MK226160	AATGGGGTAACAACAAGTGAGC
*HgNiR*-R		CAAAACCACACCACGAATCTGC
*HgNR*-F	MK226161	CACAGGTCTGACTCCCCTATTC
*HgNR*-R		ATAATCAGCCTCATTTTCATCG

### Subcellular localization assay

#### Construction of plant expression vectors

To investigate the subcellular localization of *HgNR*, *HgNiR*, *HgGS*, *HgGDH*, and *HgAS* using cDNA as a template, high fidelity polymerase KOD Neo was used to amplify the complete ORF of each gene. Using the homologous recombination method of the ClonExpress^R^ II system kit (Vazyme Biotech Co. Ltd.), target fragments were connected to the Biozyme linearized pCAMBIA2300-eGFP expression vector by *Kpn* I and *Xba* I, and the eGFP fusion expression vector was obtained. Gene-specific primers used for PCR amplification are listed in Table 4. The product was connected to the DH5α receptive cell. After coating the plate at 37°C for overnight culturing, the monoclonal antibody was selected for PCR identification. The positive clone was sequenced by LiuHe HuaDa Biotechnology (Beijing) Co., Ltd. (Beijing, China).

**Table 4:** Gene-specific primers used for analysis of expression of *HgNR*, *HgNiR*, *HgGS*, *HgGDH*, and *HgAS*.

Primer	Sequence Fragment
*HgAS*-F	**ATTTGGAGAGGACAGGGTACC**ATGTGTGGCATACTTGCTGT
*HgAS*-R	**GGTACTAGTGTCGACTCTAGA**CGAGCTAGTGATTGCAAGC
*HgGDH*-F	**ATTTGGAGAGGACAGGGTACC**ATGAATGCATTAGTTGCTAC
*HgGDH*-R	**GGTACTAGTGTCGACTCTAGA**AGCTTCCCAACCTCTGAGA
*HgGS*-F	**ATTTGGAGAGGACAGGGTACC**ATGGCGCAGATTTTGGCAC
*HgGS*-R	**GGTACTAGTGTCGACTCTAGA**GACATTCAATGCCAACTTC
*HgNiR*-F	**ATTTGGAGAGGACAGGGTACC**ATGTCATCATCATCATCGTC
*HgNiR-*R	**GGTACTAGTGTCGACTCTAGA**GCAGTCTTCTGCCTCTTC
HgNR-F	**ATTTGGAGAGGACAGGGTACC**ATGTCATCATCATCATCGTC
*HgNR*-R	**GGTACTAGTGTCGACTCTAGA**GCAGTCTTCTGCCTCTTC

The underlined portion of each primer indicates the restriction enzyme sites for *Kpn* I and *Xba* I.

#### Subcellular localization

A single colony of recombinant *A*. *tumefaciens* was cultured in YEB medium containing 50 mg/mL kanamycin and 50 mg/mL rifampicin and grown overnight at 28°C with shaking at 250 rpm. Cultures were harvested by centrifugation at 4000 rpm for 10 min and the pellet was resuspended in 10 mM MMA buffer (MES-MgCl_2_-acetosyringone) to an OD_600_ of 1, and then incubated for 3 h at room temperature. Bacterial liquid containing the positive recombinant vector was applied onto the abaxial leaf surface of 6-week old plants using a needleless 1-mL syringe. Treated plants were allowed to stand for 48 h while infiltration proceeded. Segments of infiltrated leaves were sampled from the infected area and observed under a confocal laser scanning microscope (FluoView FV1000, Olympus, Japan) [49].

### Key N metabolism-related enzyme activities

Five enzymes were determined in this study, including NR, NiR, GS, GDH, and AS. Frozen samples were ground in liquid nitrogen and weighed as 0.5–1.0 g. All enzymes were extracted under the ice-bath and determined using the corresponding ELISA detection kit in each case (Shanghai BOYE Biology Science and Technology Co., Ltd. China), according to the instructions by the manufacturer.

### Determination of fruit growth index and soluble protein

We measured the longitudinal (cm) and transverse diameters (cm) using a Vernier caliper and weighed fruit (g) using an AL204 precision electronic balance (Sartorius AG, Germany). The number of fruit and biomass yield per plant were also recorded. Total soluble solids (TSS, %), total acid (TA, g/100 mL), and vitamin C (Vc, mg/100 mL), were measured after Liao et al. [50]. Root activity was analyzed using triphenyl tetrazolium chloride (TTC) [51], and the soluble protein content was calculated as in He et al. [52].

### Statistical analysis

Statistical analysis was performed using one-way ANOVA with the SPSS 22.0 statistical software package (SPSS Inc., Chicago, IL, USA). The significance threshold was defined as *P <* 0.05.

## Results and discussion

### Cloning and subcellular localization of gene products involved in N metabolism

Full-length CDSs of various genes amplified using cDNA prepared from total RNA isolated from seedlings of the citrus cultivar ‘Huangguogan’ were cloned and their sequences confirmed through nucleotide sequencing. All cloned cDNAs were submitted to GenBank and the accession numbers are shown in Table 5. The predicted proteins ranged between 1236 and 2709 amino acids.

**Table 5:** List of proteins and their molecular weight (M. wt.), isoelectric point (PI), CDs and protein length, hydropathicity and subcellular localization.

Index	NR	NiR	GS	GDH	AS
M. wt. (KDa)	101.32	66.38	47.89	44.43	66.31
PI	6.41	6.46	6.29	6.61	5.98
CDS length (bp)	2709	1788	1299	1236	1770
Protein length (aa)	902	595	432	411	589
Grand average of hydropathicity	-0.38	-0.358	-0.484	-0.154	-0.305
Hydropathicity	Hydrophile	Hydrophile	Hydrophile	Hydrophile	Hydrophile
Subcellular localization	cytoplasmic	plasma membrane and cytoplasmic	cytoplasmic and mitochondrial	cytoplasmic	cytoplasmic and extracellular, including cell wall

**Fig 1:**
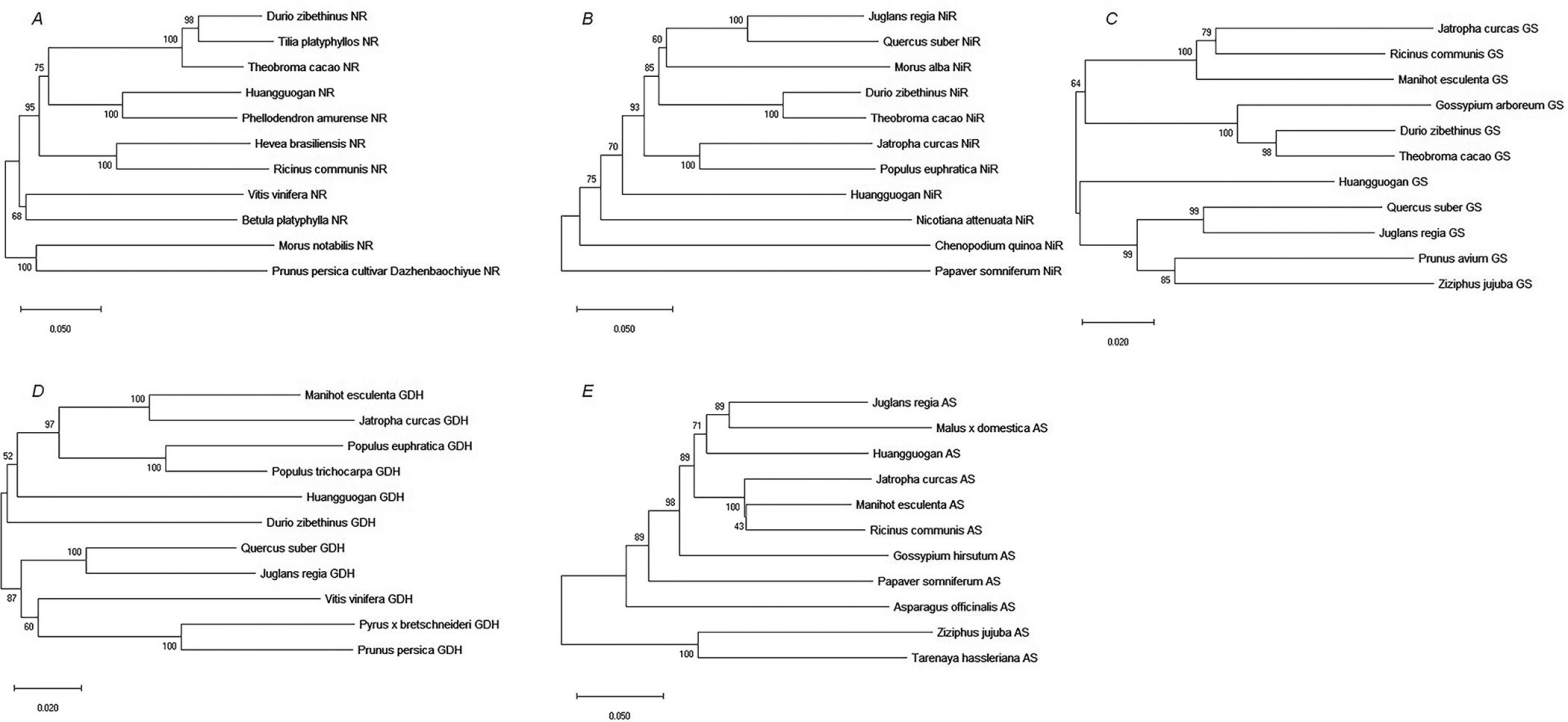
Phylogenetic tree of *HgNR*, *HgNiR*, *HgGS*, *HgGDH*, and *HgAS* genes. NR:Nitrate reductase (A), NiR:nitrite reductase (B), GS:glutamine synthetase (C), GDH:glutamate dehydrogenase (D), and AS:asparagine synthetase (E). Note: The number of nodes is bootstrap value, and the number of branches is the evolutionary distance. Sequences shown are from NCBI sequence database. Sequences of NR, NiR, GS, GDH, and AS are referred to in S1 Table.

The molecular weight of the predicted proteins ranged between 44.43 and 101.32 KDa, while their PI ranged from 5.98 to 6.61 (Table 5). Analysis and prediction of transmembrane structure and signal peptide showed that NR, NiR, GS, GDS, and AS proteins did not have a transmembrane domain or a signal peptide. It was presumed that NR, NiR, GS, GDS, and AS proteins were non-secretory, hydrophilic proteins. Phylogenetic tree analysis was applied to determine the phylogenetic positions of *HgNR*, *HgNiR*, *HgGS*, *HgGDH*, and *HgAS* from the cultivar ‘Huangguogan’ in relation to those of 10 different species. All *HgNR*, *HgNiR*, *HgGS*, *HgGDH*, and *HgAS* members in the listed species fell into two distinct groups (Fig 1).

**Fig 2:**
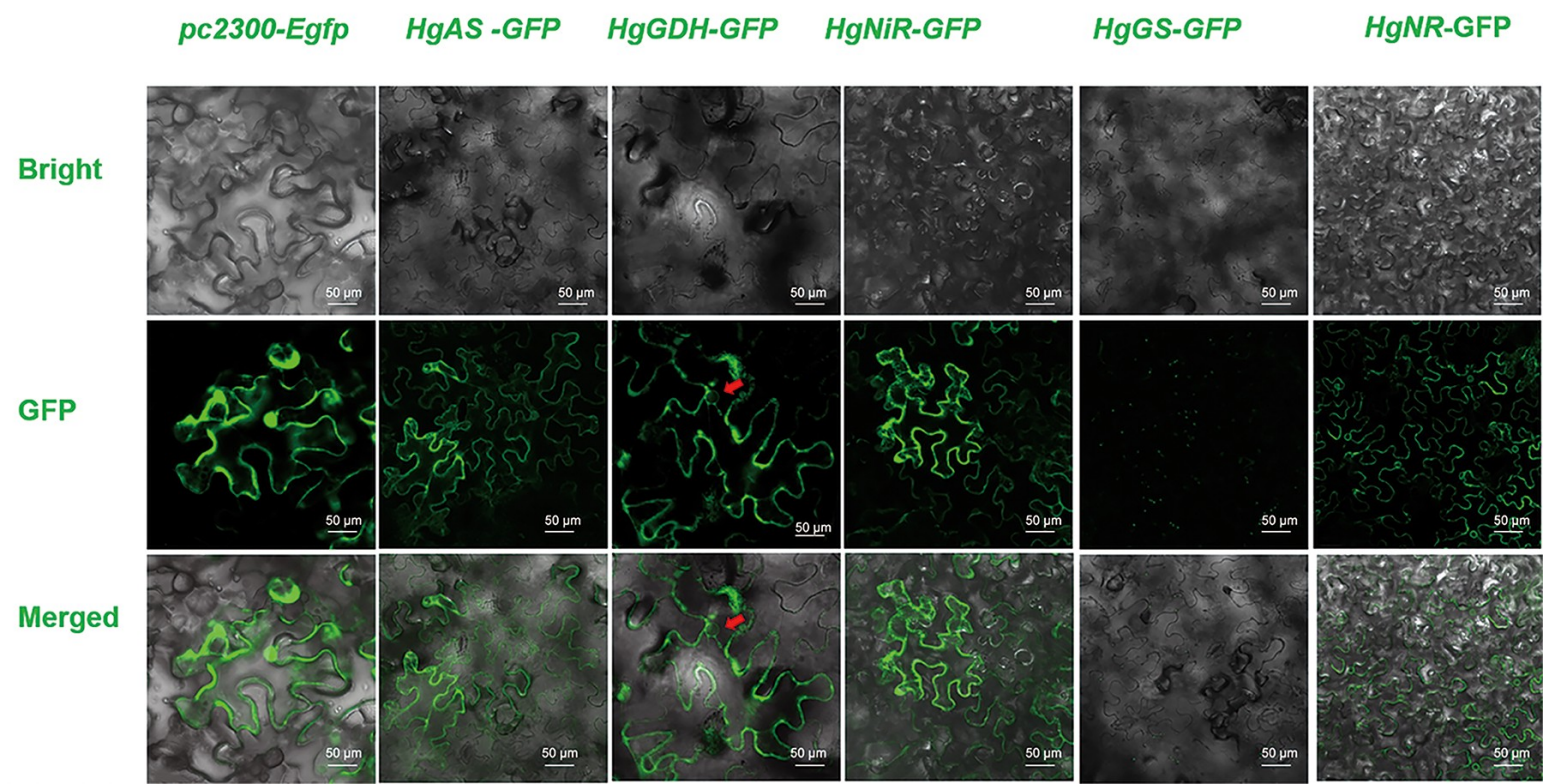
Confocal images showing fluorescence signals from *Agrobacterium*-infiltrated leaf epidermal cells. *Nicotiana benthamiana* leaves were agroinfiltrated with *HgNR*-GFP, *HgNiR*-GFP, *HgGS*-GFP, *HgGDH*-GFP, and *HgAS*-GFP.

Subcellular localization of *HgNR* was predicted in the cytoplasm; *HgNiR* was predicted to be localized in the plasma membrane and the cytoplasm, while *HgGS* was predicted to be localized in the cytoplasm and in mitochondria; *HgGDH* was found to be localized in the cytoplasm, and, lastly, *HgAS* was found to be localized in the cytoplasm and extracellularly, including the cell wall. In this study, in the tobacco cells that expressed the contrast vector pc2300-Egfp protein, fluorescence was distributed in the nucleus, the cytoplasm, and the cell membrane, but for the fusion expression of *HgNR*-GFP, fluorescence was mainly distributed in the cytoplasm and the plasma membrane. In the case of the fusion expression of *HgNiR*-GFP, fluorescence was mainly distributed in the tobacco plasma membrane and in the cytoplasm. In turn, for the fusion expression of *HgGS*-GFP, fluorescence was found mainly distributed in the cytoplasm and the mitochondria, whereas for the fusion expression of *HgGDH*-GFP, fluorescence was mainly distributed in the cytoplasm and plasma membrane. Lastly, for the fusion expression of *HgAS*-GFP, fluorescence was mainly distributed in the plasma membrane and in the cytoplasm (Fig 2).

### Effects of N application rate on the expression of N metabolism-related genes

**Fig 3:**
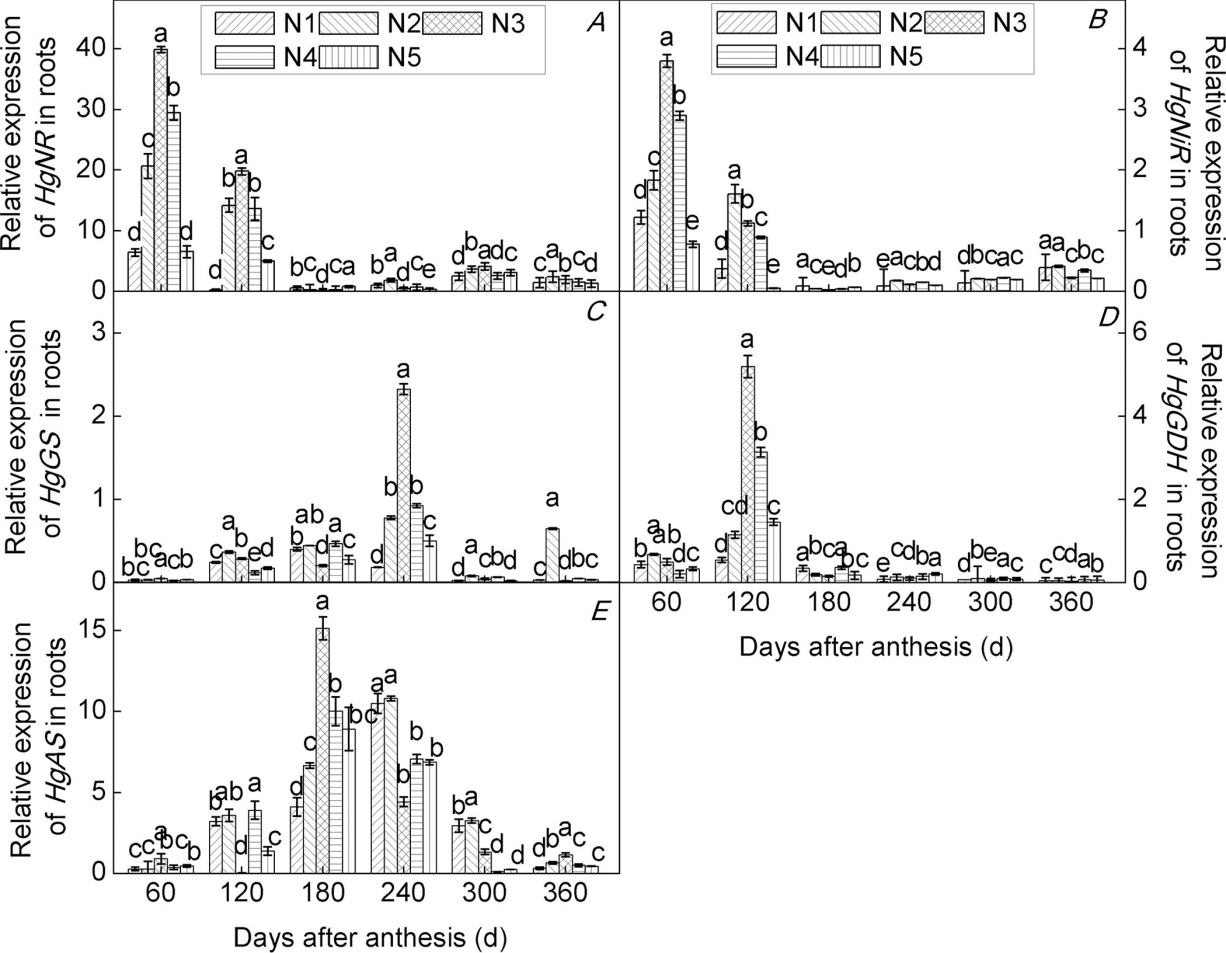
Relative gene expression in roots of the citrus cultivar ‘Huangguogan’. *HgNR* (nitrate reductase, A), *HgNiR* (nitrite reductase, B), *HgGS* (glutamine synthetase, C), *HgGDH* (glutamate dehydrogenase, D), and *HgAS* (asparagine synthetase, E). N_1_: 0, N_2_: 1.36, N_3_: 1.81, N_4_: 2.26, N_5_: 2.72 kg/year of N fertilizer [CO(NH_2_)_2_, N ≥ 46.67%].

**Fig 4:**
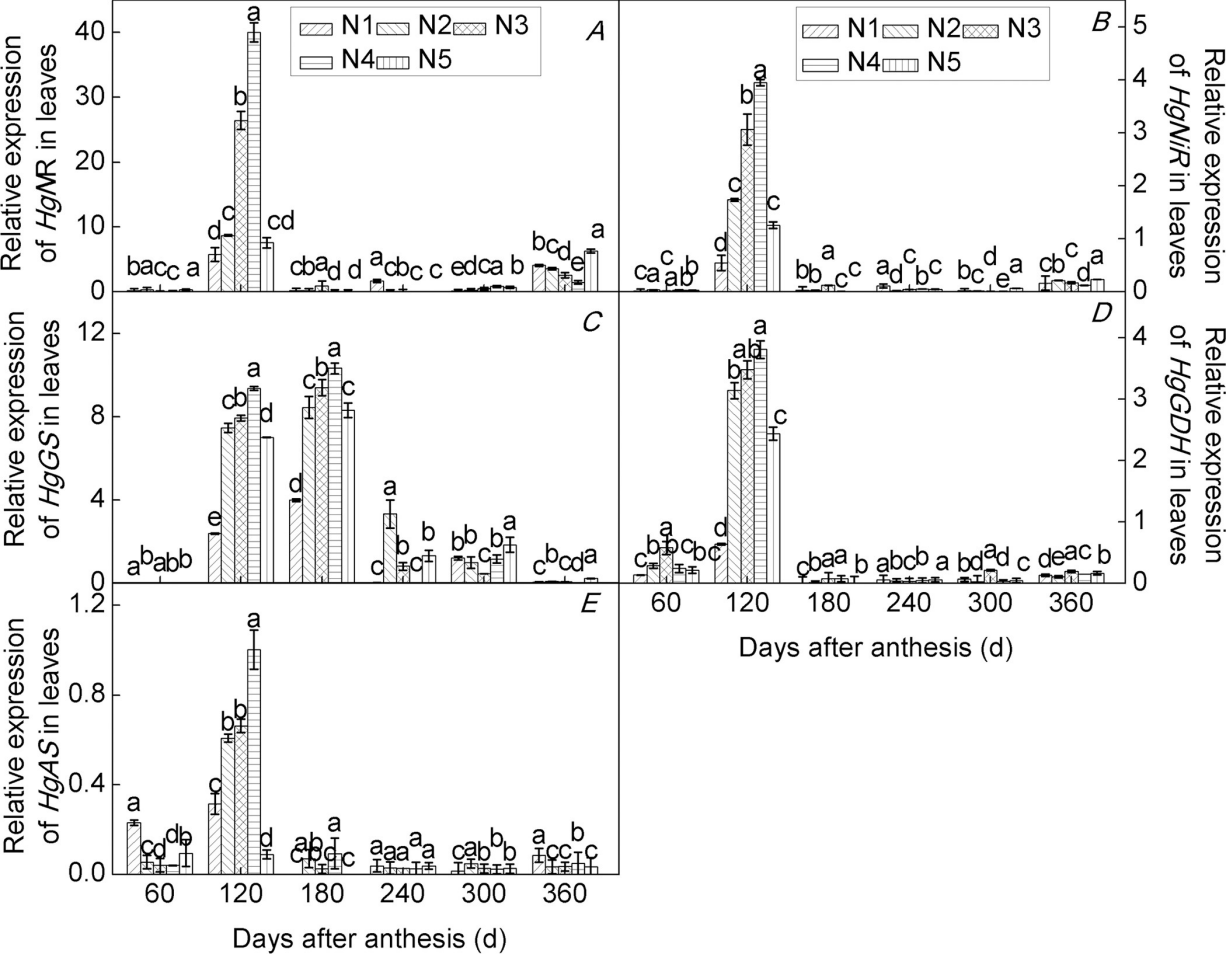
Relative gene expression in leaves of the citrus cultivar ‘Huangguogan’. *HgNR* (nitrate reductase, A), *HgNiR* (nitrite reductase, B), *HgGS* (glutamine synthetase, C), *HgGDH* (glutamate dehydrogenase, D) and *HgAS* (asparagine synthetase, E). N_1_: 0, N_2_: 1.36, N_3_: 1.81, N_4_: 2.26, N_5_: 2.72 kg/year of N fertilizer [CO(NH_2_)_2_, N ≥ 46.67%].

**Fig 5:**
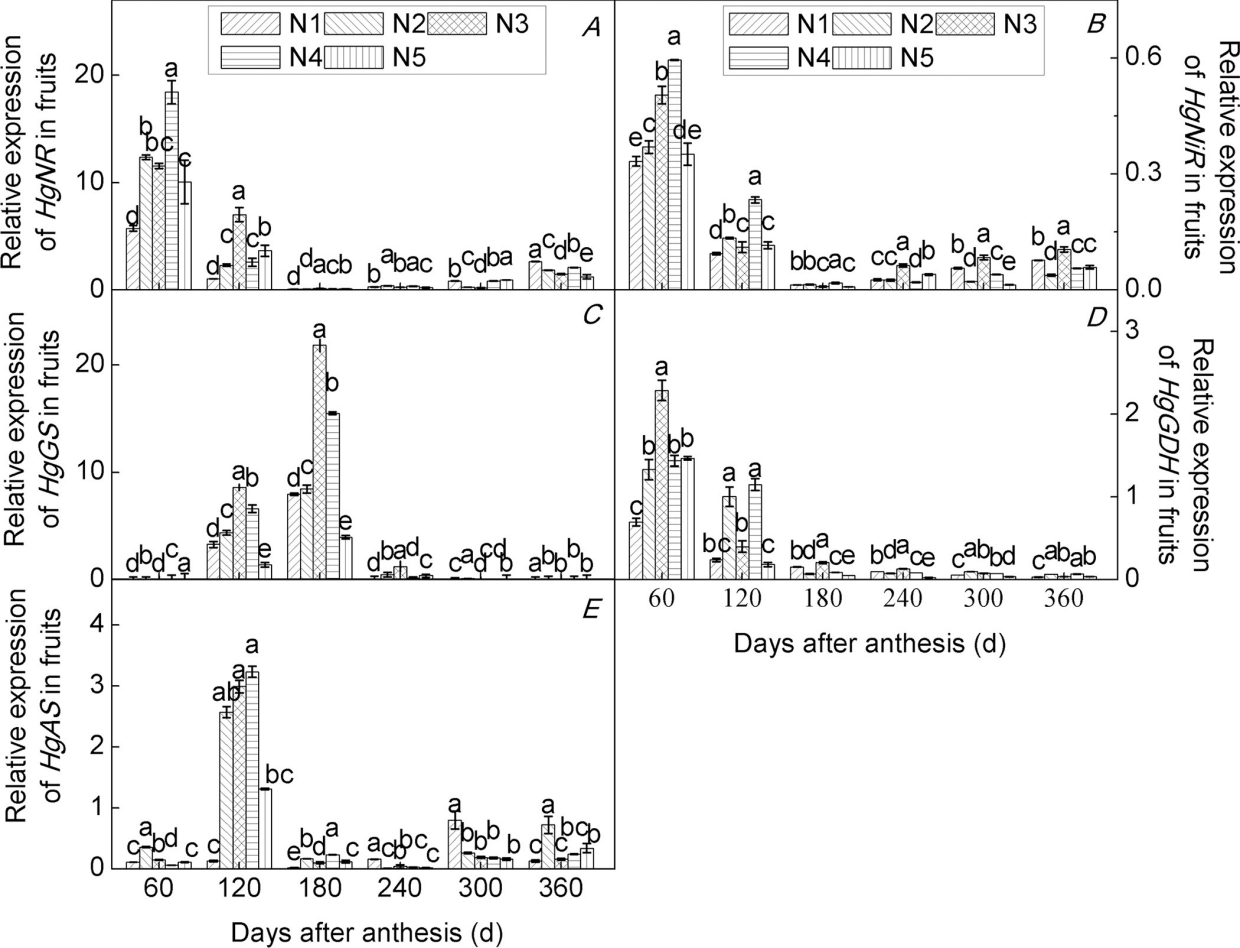
Relative gene expression in fruits of the citrus cultivar ‘Huangguogan’. *HgNR* (nitrate reductase, A), *HgNiR* (nitrite reductase, B), *HgGS* (glutamine synthetase, C), *HgGDH* (glutamate dehydrogenase, D) and *HgAS* (asparagine synthetase, E) N_1_: 0, N_2_: 1.36, N_3_: 1.81, N_4_: 2.26, N_5_: 2.72 kg/year of N fertilizer [CO(NH_2_)_2_, N ≥ 46.67%].

The expression of the key genes of N metabolism in plants is closely related to plant growth and development and can be used as a basis for exploring the nutrient requirements for fruit-tree growth [53]. In the present study, we investigated the expression of *HgNR*, *HgNiR*, *HgGS*, *HgGDH*, and *HgAS* genes in roots (Fig 3), leaves (Fig 4), and fruits (Fig 5) of seedlings of the citrus cultivar ‘Huangguogan’ at different developmental stages under five N application rates.

NR and NiR has been reported for various organs in citrus, but it is probably mainly expressed in the leaves [5]. GDH transcripts have been shown to exist in the leaves of tea and peach plants [36,54]. Lastly, a previous study showed that AS was expressed in leaves, but that it was predominantly expressed in fruit tissues [5]. In the present study, the *HgNiR*, *HgGS*, *HgGDH*, and *HgAS* genes were expressed in roots (Fig 3), leaves (Fig 4), and fruits (Fig 5) of citrus cultivar ‘Huangguogan’. The expression of the *HgNR* gene increased significantly with increasing N rates. Similarly, N significantly enhanced the transcription levels of *HgNiR*, *HgGS*, *HgGDH*, and *HgAS* genes, indicating that the genes are regulated in response to N fertilizer rate [55–57]. Previous studies have shown that the expression of nitrate assimilation genes is controlled by nitrate content in plants. For example, salt-stress leads to a reduction in nitrate transport by the roots, thus causing a strong downregulation of NR and NiR [58]. In the present study the transcription levels of *HgNR* and *HgNiR* genes were significantly reduced by excess N (i.e., 2.26 or 2.72 kg N/year) (Figs 3–5). We consider that the excessive application of N fertilizer resulted in a reduction in nitrate transport by roots, and consequently a reduction in nitrate transported to the leaves and fruits, leading to the downregulation of *HgNR* and *HgNiR* genes in roots, leaves, and fruits. Similarly, reportedly GS was downregulated under all stress conditions [58], and we found that the transcription levels of the *HgGS* gene were significantly reduced by excess N (i.e., 2.26 or 2.72 kg N/year). This may be due to excess N promoting plant uptake of NH4^+^, thus accumulating large amounts of NH_4_^+^ in the plant and inhibiting GS [59]. Skopelitis et al. [60] reported that the expression of GDH increased in tobacco under salt-stress conditions. However, we found that the *HgGDH* gene was downregulated under excessive N, suggesting that different stress conditions lead to different reaction mechanisms. Similarly, the expression of the *AS* gene was different under different stress conditions. For example, *AS* was upregulated under salt, osmotic, and heat stresses, while it was downregulated under cold stress [58]. In the present study, the *HgAS* gene was downregulated under excessive N (Figs 3–5).

Nitrogen metabolism enzyme-encoding genes exhibited different response mechanisms to N rates and application time among the different trees and organs of the same tree [61]. We consistently observed differences in the expression of key enzymes of N metabolism in different organs of young trees of the citrus cultivar ‘Huangguogan’. For example, maximum transcription levels of *HgNR* in roots of N_2_-, N_3_-, N_4_-, and N_5_-treated trees were 220.0%, 517.7%, 356.0%, and 1.6% higher than those observed in N_1_ trees, respectively(Fig 3). Similarly, in leaves of N_2_-, N_3_-, N_4_-, and N_5_-treated trees, maximum transcription levels of *HgNR* increased by 51.5%, 360.7%, 597.5%, and 31.7% in relation to that found in N_1_ trees, respectively(Fig 4). On the other hand, in fruits, maximum transcription level of *HgNR* was 115.4%, 101.3%, 221.3%, and 75.4% higher in N_2_-, N_3_-, N_4_-, and N_5_-treated trees, respectively, than in the fruits of N_1_ trees(Fig 5). Similarly, the transcription levels of *HgNiR*, *HgGS*, *HgGDH*, and *HgAS* genes differed among the organs of the cultivar ‘Huangguogan’. This indicates interactions among the expression of N metabolism-related genes in different organs of the cultivar ‘Huangguogan’, which agrees with a previous report [62].

Short- and long-term exposure to changing environments leads to changes in plant gene expression [63]. The pattern of gene expression under low N (i.e., 1.36 kg/year) provided a new insight into the phenomenon of plant acclimation to N fertilization and can be considered as a general response to soil N conditions. However, transcriptional changes under high-N conditions (2.26 or 2.72 kg per year) were more specific after the growth cycle(Figs 3–5). Investigation of the expression level of genes encoding enzymes and proteins involved in N transport and metabolism is a crucial step in gaining a clearer understanding of the mechanisms underlying plant responses to N supply. This understanding may help breeders develop citrus cultivars with higher NUE and agronomists in improving crop fertilization and production management [64]. Despite slight differences in expression level among *HgNR*, *HgNiR*, *HgGS*, *HgGDH*, and *HgAS* genes, and the corresponding enzyme activity levels in different organs at different stages of growth in young trees of the citrus cultivar ‘Huangguogan’ grown under different N-application rates, we concluded that, as the root is permanently in direct contact with the soil, it directly affects the process of nutrients entering the plant from the soil. Therefore, we suggest that root gene expression at different growth stages can be used as a reference to determine N-application rates for the citrus cultivar ‘Huangguogan’.

### Effects of N application rate on key enzyme activities of N metabolism

**Fig 6:**
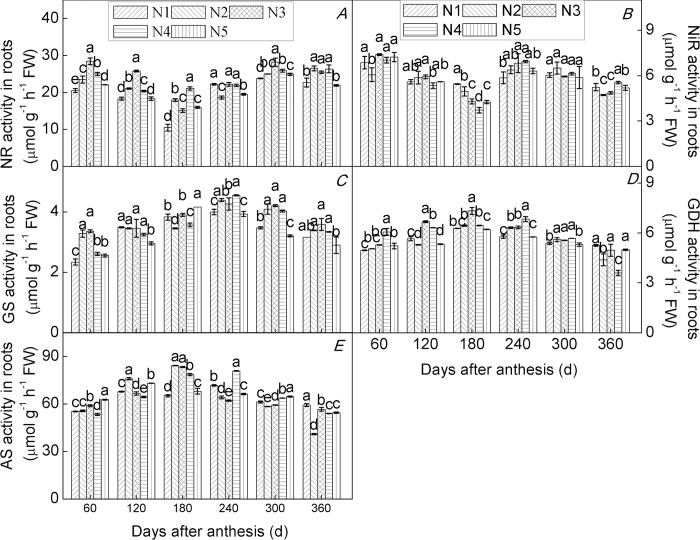
Relative enzyme activity in roots of the citrus cultivar ‘Huangguogan’. NR (Nitrate reductase, A), NiR (nitrite reductase, B), GS (glutamine synthetase, C), GDH (glutamate dehydrogenase, D) and AS (asparagine synthetase, E). N_1_: 0, N_2_: 1.36, N_3_: 1.81, N_4_: 2.26, N_5_: 2.72 kg N/ year [CO(NH_2_)_2_, N ≥ 46.67%].

**Fig 7:**
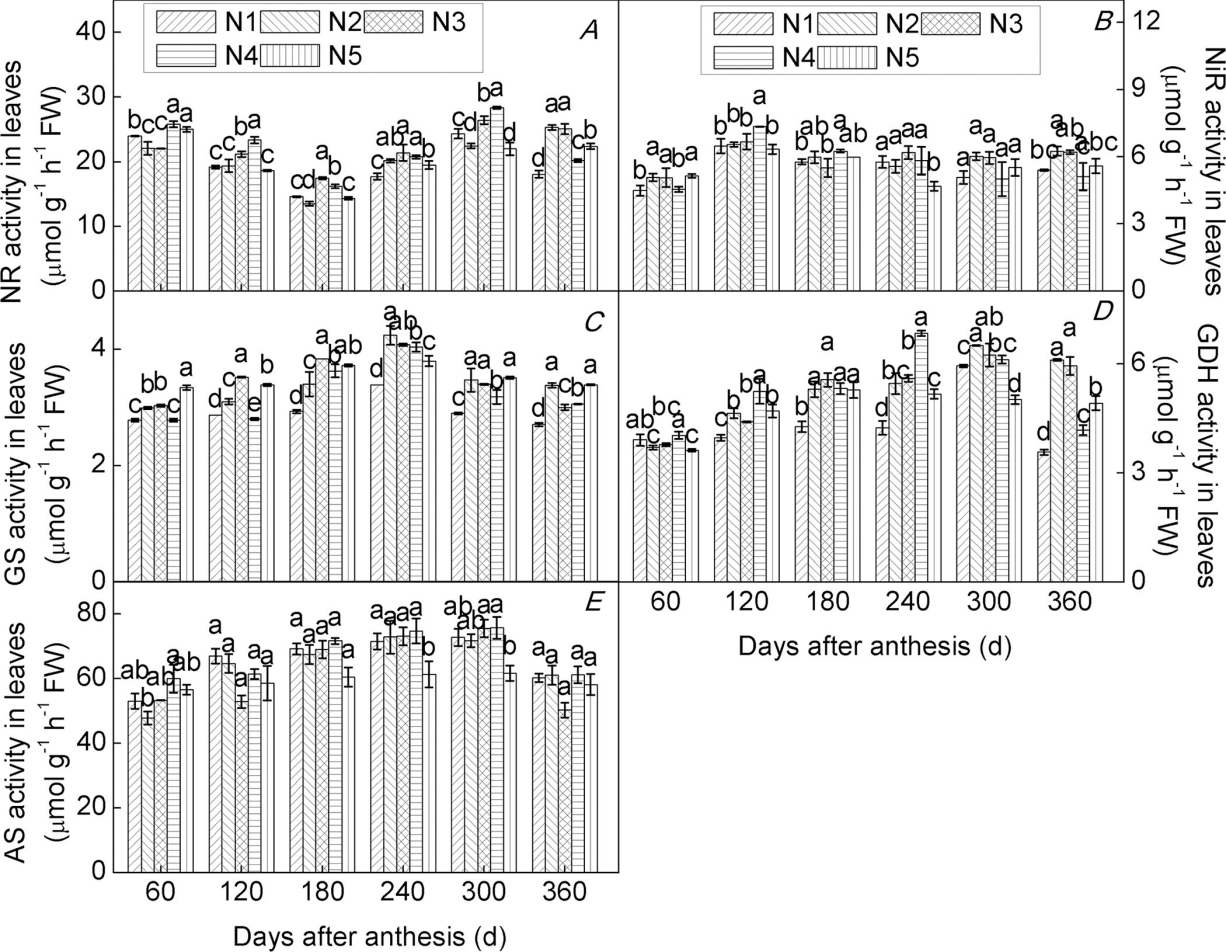
Relative enzyme activity in leaves of the citrus cultivar ‘Huangguogan’. NR (Nitrate reductase, A), NiR (nitrite reductase, B), GS (glutamine synthetase, C), GDH (glutamate dehydrogenase, D) and AS (asparagine synthetase, E). N_1_: 0, N_2_: 1.36, N_3_: 1.81, N_4_: 2.26, N_5_: 2.72 kg N/ year [CO(NH_2_)_2_, N ≥ 46.67%].

**Fig 8:**
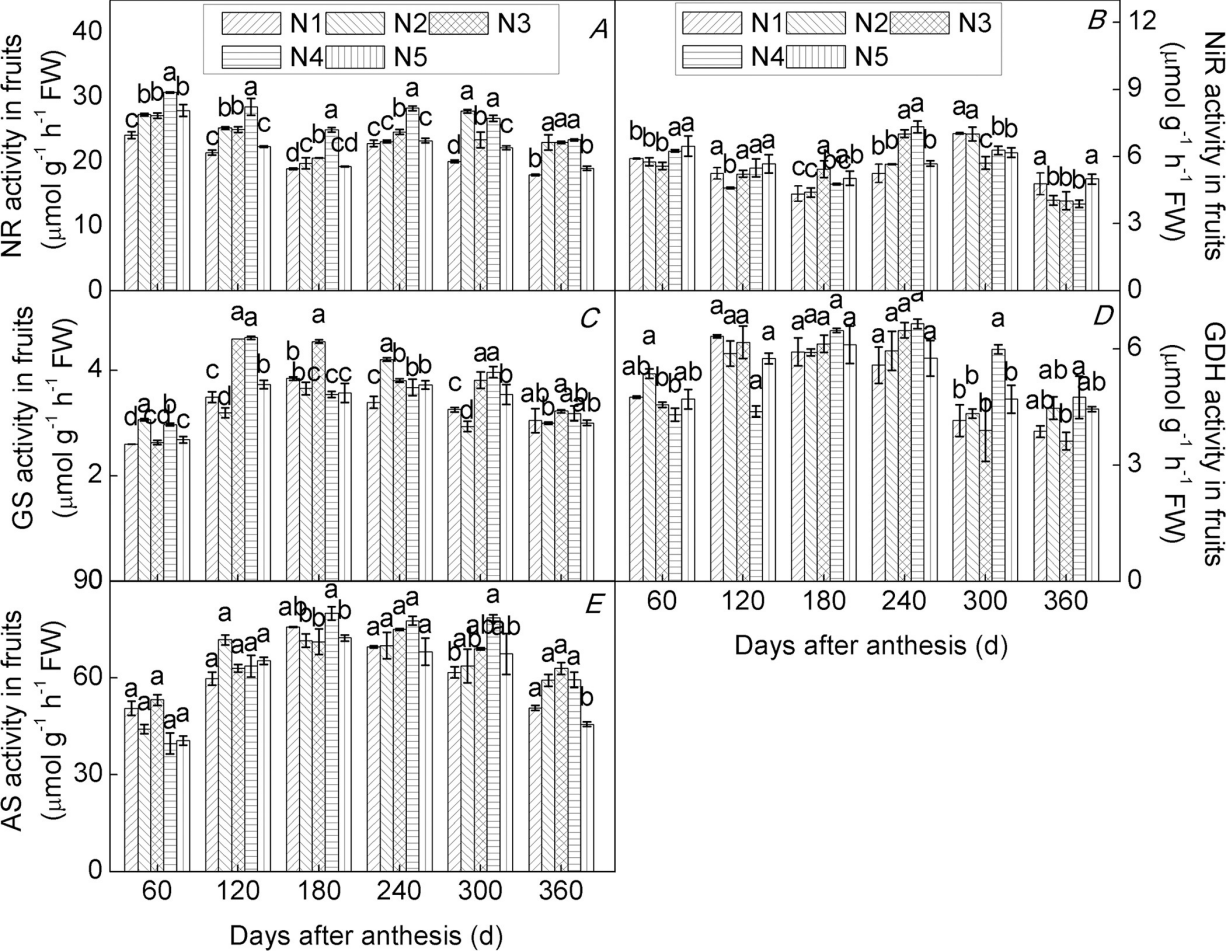
Relative enzyme activity in fruits of the citrus cultivar ‘Huangguogan’. NR (Nitrate reductase, A), NiR (nitrite reductase, B), GS (glutamine synthetase, C), GDH (glutamate dehydrogenase, D) and AS (asparagine synthetase, E) s. N_1_: 0, N_2_: 1.36, N_3_: 1.81, N_4_: 2.26, N_5_: 2.72 kg N/ year [CO(NH_2_)_2_, N ≥ 46.67%].

Enzyme activity is subject to regulation at the level of gene expression, and plants respond to changes in soil N by adjusting the expression of genes involved in N metabolism [33,65]. In the present study, we found that all enzyme activities monitored varied significantly and differentially among roots (Fig 6), leaves (Fig 7), and fruits (Fig 8) of trees of the citrus cultivar ‘Huangguogan’ with N supply, over the experimental period. The trends in activities of NR, NiR, GS, GDS, and AS were consistent with those of *HgNR*, *HgNiR*, *HgGS*, *HgGDH*, and *HgAS* transcription, respectively. Cruz et al. [66] reported that activities of NR, GS, and GDH were considerably lower under low-nitrate supply than under high-nitrate supply in cassava plants. Similarly, we found that an appropriate increase in N fertilizer can significantly increase the activities of NR, NiR, GS, GDS, and AS, in agreement with previous studies that have demonstrated that N supply can increase the activity of key enzymes involved in N metabolism [67,68]. For example, sufficient NR activity is a prerequisite for optimal utilization of soil N [20]. In this sense, it has been found that the root tip (0–2 cm) is the most active part of the root for N uptake [69].

Sulpice et al. [70] found that low N caused a decrease in nitrate reductase activity. We measured maximum NR activity in root tips of N_2_-, N_3_-, N_4_-, and N_5_-treated trees and found that it was 5.0%, 18.5%, 8.9%, and 4.7% higher than in N_1_-treated trees, respectively(Fig 6). Similarly, in leaves of N_2_-, N_3_-, N_4_-, and N_5_-treated trees, maximum NR activity increased by 13.8%, 20.7%, 17.1%, and 9.9%, compared to that found in N_1_-treated trees(Fig 7), while maximum NR activity was 38.7%, 16.6%, 33.2%, and 10.4% higher in fruits of N_2_-, N_3_-, N_4_-, and N_5_-treated trees, respectively, than in fruits of N_1_-treated trees(Fig 8). Similarly, maximum levels of activity for NiR, GS, GDH, and AS increased initially and then decreased with further increases in N-application rate. These findings demonstrated that N directly and positively affected all measured parameters of N metabolism in roots, leaves, and fruits of young trees of ‘Huangguogan’. However, all enzyme activities significantly decreased under excess N supply (i.e. 2.26 or 2.72 kg N/year), indicating that excess N inhibited root, leaf, and fruit normal N metabolism, thereby limiting the normal physiological function associated with N. This is consistent with previous studies [71]. Therefore, the application of N fertilizer should be controlled below 2.12 kg/year, which has a more beneficial effect on N metabolism, according to our findings.

### Effects of N application on growth and fruit quality of ‘Huangguogan’

**Fig 9:**
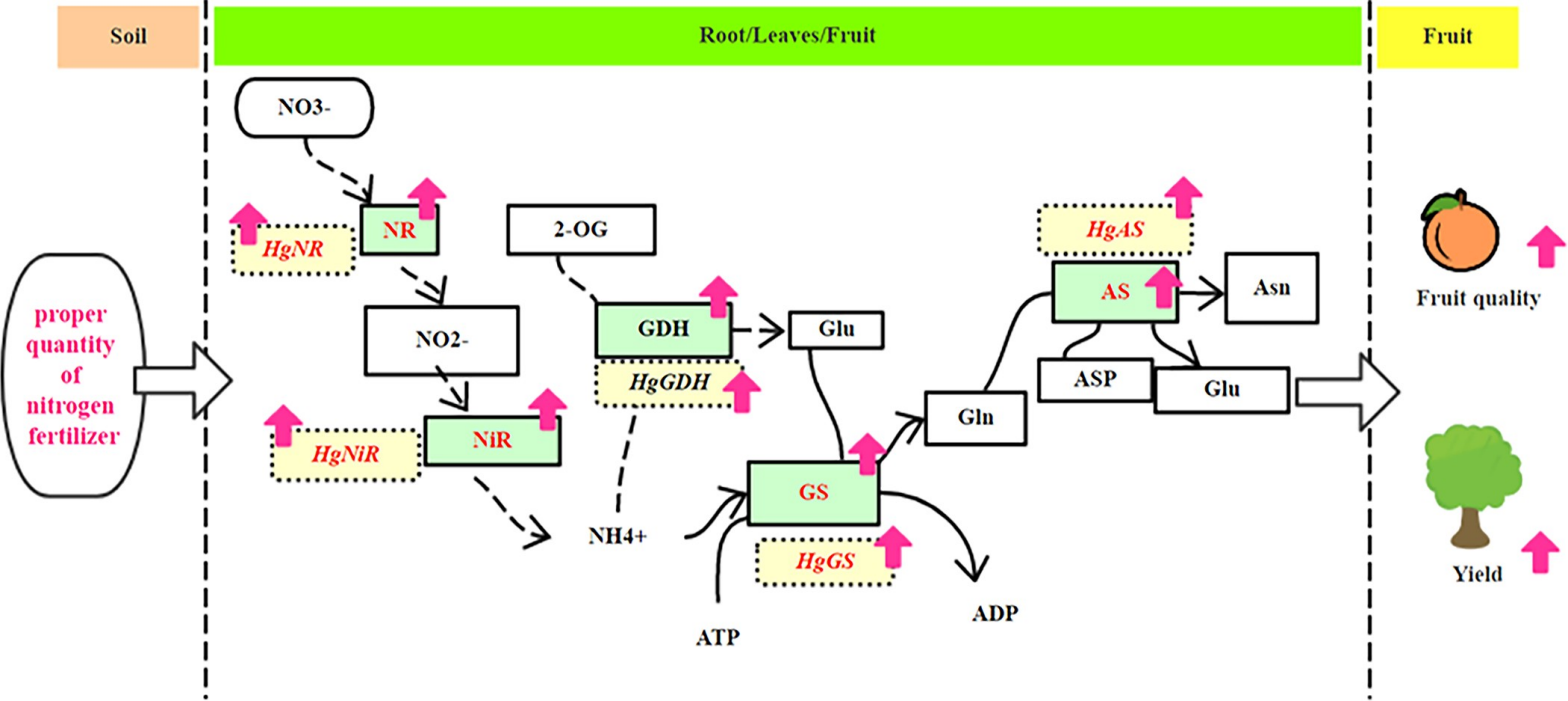
Graphical abstract. The solid and dashed lines denote the pathways of inorganic N metabolism in the citrus cultivar ‘Huangguogan,’ and the red upward arrow indicates that the relevant indicators were upregulated under adequate N supply.

Numerous studies have shown that the application of N fertilizer can significantly improve the quality of citrus fruits [4,5], and that a N limitation frequently reduces growth and yield [72]. In the present study, we found that fruit shape indices first increased, then decreased with increased N supplementation, but this change was not significant, suggesting that N changed fruit size but had little effect on fruit shape, moreover, increasing N supply initially led to increased yield, single fruit weight, TSS, TA, Vc, and root activity (Table 6), but that these parameters all decreased significantly when N concentration exceeded 1.81 kg/year (i.e. in N_4_- and N_5_-treated plants). These results indicate that excess N supply exerts a significant inhibitory effect, particularly affecting fruit quality, yield [73], and root growth [74]. Recent studies have shown a significant correlation between N metabolism and tobacco leaf quality [71]. N metabolism mainly involves a series of continuous processes that convert mineral N into organic N (NO_3_^-^→NO_2_^-^→NH_4_^+^ → glutamine → glutamic acid → amino acid → protein) [75]. Evans [76] found that the rate of CO_2_ assimilation in leaves correlated positively with leaf N level, but the correlation decreased significantly under supraoptimal leaf-N content, indicating that excess N inhibited N metabolism and assimilation, and disrupted crop physiological functions [77]. In the present study, leaf N and soluble protein content were similar to the activity of N metabolizing enzymes, which increased initially and then decreased with further increases in N supply. Our study indicated that the appropriate application of N fertilizer can promote the accumulation of protein in the plant. By increasing the expression of genes encoding key N metabolism-related enzyme activities, N reduction and assimilation are improved, and the physiological functions of roots, leaves, and fruits of citrus plants can be improved. This leads to the improvement of single fruit weight and intrinsic quality, while excess N leads to deleterious effects on those variables, which may be due to the decline in the activities of N metabolism enzymes leading to a significant decline in sugar metabolism enzymes in crop leaves (Fig 9). This would affect the synthesis and transformation of amino acids, and ultimately inhibit N metabolism and disrupt N absorption and translocation. Overall, this would cause physiological processes associated with N to be hampered, resulting in a significant reduction of yield and fruit quality [77].

**Table 6:** Effects of different N levels on the cross section (cm), the vertical diameter (cm), the fruit shape index, yield (kg/plant), single fruit weight (g), total soluble solids (TSS, %), total acid (TA, g/100 mL), vitamin C (Vc, mg/100 mL), soluble protein in fruit, root activity, soluble protein in roots, leaf N, and soluble protein in leaves of ‘Huangguogan’. Data are means ± SD of five replicates. Different lowercase letters indicate significant differences (*P* < 0.05) among treatments.

index	N1	N2	N3	N4	N5
The cross section	6.38±0.24 a	6.78±0.31 a	6.58±0.12 a	6.64±0.2 a	6.5±0.32 a
The vertical diameter	6.06±0.31 a	6.46±0.32 a	6.4±0.49 a	6.58±0.16 a	5.99±0.04 b
The fruit shape index	0.94±0.02 a	0.96±0.06 a	0.97±0.06 a	0.99±0.01 a	0.92±0.05 a
Yield	103.57±2.22 d	144.17±4.71 b	158.12±10.95 a	136.47±4.02 b	127.87±4.17 bc
Single fruit weight	141.83±1.5 a	150.24±2.89 c	154.2±2.55 a	140.78±2.43 c	134.22±2.42 b
TSS	12.53±0.38 a	12.93±1.18 a	13.09±0.57 a	12.37±1.17 a	12.13±1.1 a
TA	0.92±0.04 a	0.71±0.03 b	0.69±0.01 b	0.67±0.04 b	0.66±0.03 b
Vc	38.04±0.61 b	38.85±0.85 b	41.48±0.25 a	38.97±0.56 b	38.81±0.46 b
Soluble protein in fruit	49.84±3.58b	23.64±1.3c	50.7±4.75b	60.83±6.39a	45.26±3.17b
Root activity	9.57±1.47 a	10.32±2.36 a	7.84±2.2 a	8.56±3.23 a	8.16±2.29 a
Soluble protein in roots	50.26±0.71e	65.63±0.49c	75.71±3.71a	62.06±1.78b	58.14±0.68d
Leaf N	2.56±0.02d	2.73±0.04cd	2.85±0.04bc	3.76±0.02a	3.08±0.02b
Soluble protein in leaves	64.95±3.71b	49.44±2.53c	99.04±3.86a	22.47±1.53e	32.25±2.91d

## Conclusions

Increased N fertilizer significantly enhanced the activity of key N metabolism enzymes (NR, NiR, GS, GDH, and AS) and the expression of their related genes (*HgNR*, *HgNiR*, *HgGS*, *HgGDH*, and *HgAS*) in roots, leaves, and fruits. Increasing N rates also improved root activity, leaf N content, soluble protein, and ‘Huangguogan’ fruit quality. However, excess N supply exerted a significant inhibitory effect on normal in root, leaf, and fruit N metabolism, thereby reducing ‘Huangguogan fruit quality and yield. Therefore, we recommend 1.81 kg N/year as the optimal N application rate for young trees of ‘Huangguogan’.

## Supporting information

S1 Table List of the NR, NiR, GS, GDH, and AS sequences used in phylogenetic tree analysis. (XLS)
