# Correction: Effect of nitrogen supply on nitrogen metabolism in the citrus cultivar ‘Huangguogan’

**DOI:** 10.1371/journal.pone.0216639

**Published:** 2019-05-02

**Authors:** Ling Liao, Tiantian Dong, Xinya Liu, Zhixiang Dong, Xia Qiu, Yi Rong, Guochao Sun, Zhihui Wang

Supporting Information file S1 Table contains mistakenly published data. The incorrectly published data are presented on Sheet 1 in the table labeled, “Table S2. List of the NR, NiR, GS, GDH, and AS sequences used in phylogenetic tree analysis.” These data are not relevant to the current study and were previously published as S2 Table by Xu et al. [2]. The authors used the previously published Supporting Information file as a table template but did not delete the data originally included in the previously published Supporting Information file prior to the publication of the current study. The data presented on Sheet 2 in the table labeled, “S1 Table. List of the NR, NiR, GS, GDH, and AS sequences used in phylogenetic tree analysis,” are correct and complete.

Please view the correct S1 Table below.

## Supporting information

S1 Table List of the NR, NiR, GS, GDH, and AS sequences used in phylogenetic tree analysis. (XLS)

## References

[1] Liao L, Dong T, Liu X, Dong Z, Qiu X, Rong Y, et al (2019) Effect of nitrogen supply on nitrogen metabolism in the citrus cultivar ‘Huangguogan’. PLoS ONE 14(3): e0213874 10.1371/journal.pone.0213874 30897177 PMC6428318

[2] Wan L, Ma J, Wang N, Wang D, Xu G (2013) Molecular Cloning and Characterization of Different Expression of *MYOZ2* and *MYOZ3* in Tianfu Goat. PLoS ONE 8(12): e82550 10.1371/journal.pone.0082550 24367523 PMC3867352

